# Comparison of functional outcomes following early and delayed arthroscopic repair for traumatic and non-traumatic rotator cuff injuries

**DOI:** 10.1186/s13018-024-04858-x

**Published:** 2024-06-21

**Authors:** Aixin Liu, Baorui Zhang, Tong Lai, Mingxing Wang, Gongyi Wu, Shilin Liu, Tao Zhang

**Affiliations:** 1 Department of Orthopaedic Surgery, Lu’an Hospital of Traditional Chinese Medicine Affiliated of Anhui University of Traditional Chinese Medicine, Lu’an, Anhui 237000 China; 2 Department of Rehabilitation, Lu’an Hospital of Traditional Chinese Medicine Affiliated of Anhui University of Traditional Chinese Medicine, Lu’an, Anhui 237000 China; 3 Department of Oncology, Lu’an Hospital of Traditional Chinese Medicine Affiliated of Anhui, University of Traditional Chinese Medicine, Lu’an, Anhui 237000 China; 4 Department of Neurocritical Care Unit, The First Affiliated Hospital of USTC, Division of Life Sciences and Medicine, University of Science and Technology of China, Hefei, Anhui 230001 China; 5 Department of Orthopaedic Surgery, Lu’an City Hospital of Traditional Chinese Medicine, Lu’an, Anhui 237000 China

**Keywords:** Rotator cuff injury, Rotator cuff repair, Timing, Function, Outcomes

## Abstract

**Background:**

The effects of the timing of surgical repair on the outcomes of traumatic and non-traumatic rotator cuff injuries (RCI) remain elusive. Thus, this study aimed to compare differences in outcomes following the repair of traumatic and non-traumatic RCI at varying time points.

**Methods:**

The study population comprised 87 patients with traumatic and non-traumatic RCI who underwent arthroscopic rotator cuff repair and were followed up for a minimum of 6 months. Next, the trauma and the non-trauma groups were stratified into subgroups according to the time of injury (early repair: occurring within 3 months; delayed repair: occurring after 3 months). Measurements before and after surgical interventions were compared to evaluate the effect of the duration of RCI on the functional status of patients in the trauma and non-trauma groups. Primary evaluation indices included the Visual Analog Scale (VAS) pain score, American Shoulder and Elbow Surgeons (ASES) score, Constant shoulder function score, and the University of California, Los Angeles (UCLA) shoulder score. Secondary evaluation indices consisted of shoulder range of motion (ROM), postoperative rotator cuff retear rate, and incidence of joint stiffness.

**Results:**

Among the 40 patients in the trauma group, 22 underwent early repair, whereas the remaining 18 underwent delayed repair. In the non-trauma group consisting of 47 patients, 18 underwent early repair, whereas the remaining 29 underwent delayed repair. The minimum clinical follow-up time was 6 months, with an average follow-up time of 10.2 months. During postoperative follow-up, 1 and 6 patients who underwent early and delayed repair experienced re-tear in the trauma group, respectively. Contrastingly, 3 and 8 patients who underwent early and delayed repair presented with re-tear in the non-trauma group, respectively.

**Conclusion:**

Early repair of traumatic RCI yielded superior outcomes, including improved range of motion, lower pain symptoms, and lower risk of postoperative re-tears compared to delayed repair. Additionally, non-surgical treatment is recommended as the preferred approach for patients with non-traumatic RCI.

## Background

Rotator cuff injury (RCI) is a prevalent condition characterized by shoulder pain and dysfunction in adults, particularly the elderly [1–3]. Mounting evidence indicates [4] that among individuals aged over 60 years, 36% of individuals experience shoulder pain, and 16.9% of asymptomatic individuals develop shoulder tears. Notably, the causes of rotator cuff injuries are multifaceted, including intrinsic (nontraumatic) and extrinsic (traumatic) causes, and social factors. The degeneration of tendon tissue in RCI is a gradual process, exacerbated by the vulnerable location of the rotator cuff, rendering the cuff susceptible to friction and impingement, ultimately leading to partial or complete tendon tears [5]. While some patients respond to conservative treatment [6–9], those with severe tears often necessitate arthroscopic rotator cuff repair [10–12]. At present, total arthroscopic rotator cuff repair [13] is the most common surgical approach that involves single-row, double-row, or suture-bridge techniques based on tear severity [14–16]. Previous studies [17, 18] documented that patients with traumatic rotator cuff injuries generally exhibit better surgical outcomes, possibly attributable to improved tendon repair and a shorter injury-to-surgery interval. However, the influence of the duration of injury on postoperative outcomes in surgically repaired traumatic and nontraumatic patients remains to be elucidated, with the ideal timing for surgery in RCI being controversial [19, 20]. Therefore, evidence-based insights into the surgical management of traumatic and non-traumatic rotator cuff repairs are essential for achieving optimal patient outcomes.

This study aimed to compare the influence of the timing of surgical intervention on postoperative functional and clinical outcomes in patients with traumatic and non-traumatic rotator cuff injuries. We hypothesized that early repair results in greater improvements in range of motion (ROM) and clinical outcomes. Elucidating the effect of the timing of surgical intervention on postoperative outcomes of patients with rotator cuff repair with different tear etiologies may assist in preoperative counseling and shared clinical decision-making.

## Methods

### Participants

This retrospective study enrolled patients attending Lu’an Hospital of Traditional Chinese Medicine, affiliated with Anhui University of Traditional Chinese Medicine, between June 2021 and June 2023. It was approved by our Institutional Review Board, and the requirement for informed consent was waived by the committee owing to the retrospective nature of the study. All procedures were performed by the same experienced sports medicine physician and two assistants. The inclusion criteria were as follows: (1) patients aged above 18 years with a minimum follow-up duration of 6 months; (2) preoperative magnetic resonance imaging (MRI) was used for the diagnosis of rotator cuff injury; (3) patients undergoing primary arthroscopic rotator cuff repair. The exclusion criteria were: (1) comorbid shoulder osteoarthritis, shoulder instability, or fracture; (2) a history of shoulder surgery on the same side.

### Study design

**Fig. 1:**
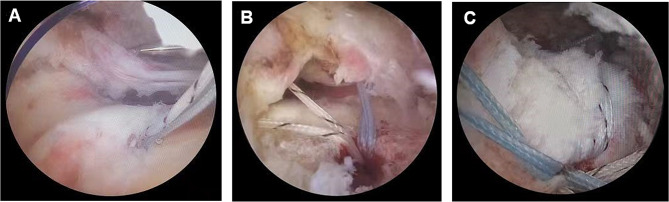
Double row anchor suture technique under arthroscope.**A**, Two anchors were successively inserted in the inner row; **B**, The suture was passed through the superordinate tendon, closed, and excess sutures were removed; **C**, The outer-row anchors were implanted, and excess sutures were cut off

The clinical data of patients were acquired from chart review, including age, gender, affected limb side, smoking status, history of hypertension and diabetes mellitus, and mode of repair. Clinical data were recorded during preoperative and postoperative follow-up. The VAS score, American Shoulder and Elbow Surgeons (ASES) score, Constant Shoulder Function score, and the University of California at Los Angeles scoring system (UCLA) were assessed via questionnaires and physical examination. Meanwhile, shoulder mobility ROM was evaluated through active movement, comprising shoulder forward flexion (FF), external rotation (ER), and internal rotation (IR). Based on MRI images and intraoperative exploration of tendon integrity and involved tendons, tear type was categorized as either total or partial, and the morphology of the acromion and the performance of acromioplasty were evaluated to select the appropriate repair modality (single-row suture, double-row suture, and suture bridge technique) (Fig. 1). Postoperative complications following rotator cuff repair were documented during clinical follow-up visits, and final outcomes, including the incidence of rotator cuff retear and joint stiffness, were recorded at the 6th month. Shoulder stiffness was defined as a forward elevation of less than 120°, external rotation of less than 30°, and internal rotation of the back of less than L3. All imaging data were blindly assessed by two senior radiologists.

### Study cohorts

Based on the history of trauma events and the presence of muscle edema in magnetic resonance images, patients were categorized into either the trauma group or the non-trauma group. Subsequently, each group was further stratified into early repair and delayed repair subgroups, resulting in a total of four groups. Within the trauma group, patients were classified into the early repair subgroup if the interval between shoulder cuff injury and surgery was within 3 months, whereas those with an interval exceeding 3 months were assigned to the delayed repair subgroup. Likewise, patients in the non-trauma group were categorized into the early repair subgroup if the duration of reported pain was less than 3 months and into the delayed repair subgroup if the duration exceeded 3 months. Comparisons were made between preoperatively and postoperative values at final follow-up within each cohort and between cohorts.

### Statistical analysis

Variables following a normal distribution were expressed as mean ± standard deviation (SD), and the normality of the data was evaluated using the Kolmogorov-Smirnov test. Normally distributed data were analyzed using the t-test, whilst non-normally distributed data were compared using the Mann-Whitney U-test and expressed as the median and interquartile range (IQR). Categorical variable comparisons were performed using the chi-square test (X²) or Fisher’s exact test, depending on the distribution of the data. Statistical analyses were performed using SPSS Statistics 24.0 (Version 20.0, IBM Corporation, Armonk, NY), and differences were considered statistically significant at *p* < 0.05.

## Results

### Patient selection

**Fig. 2:**
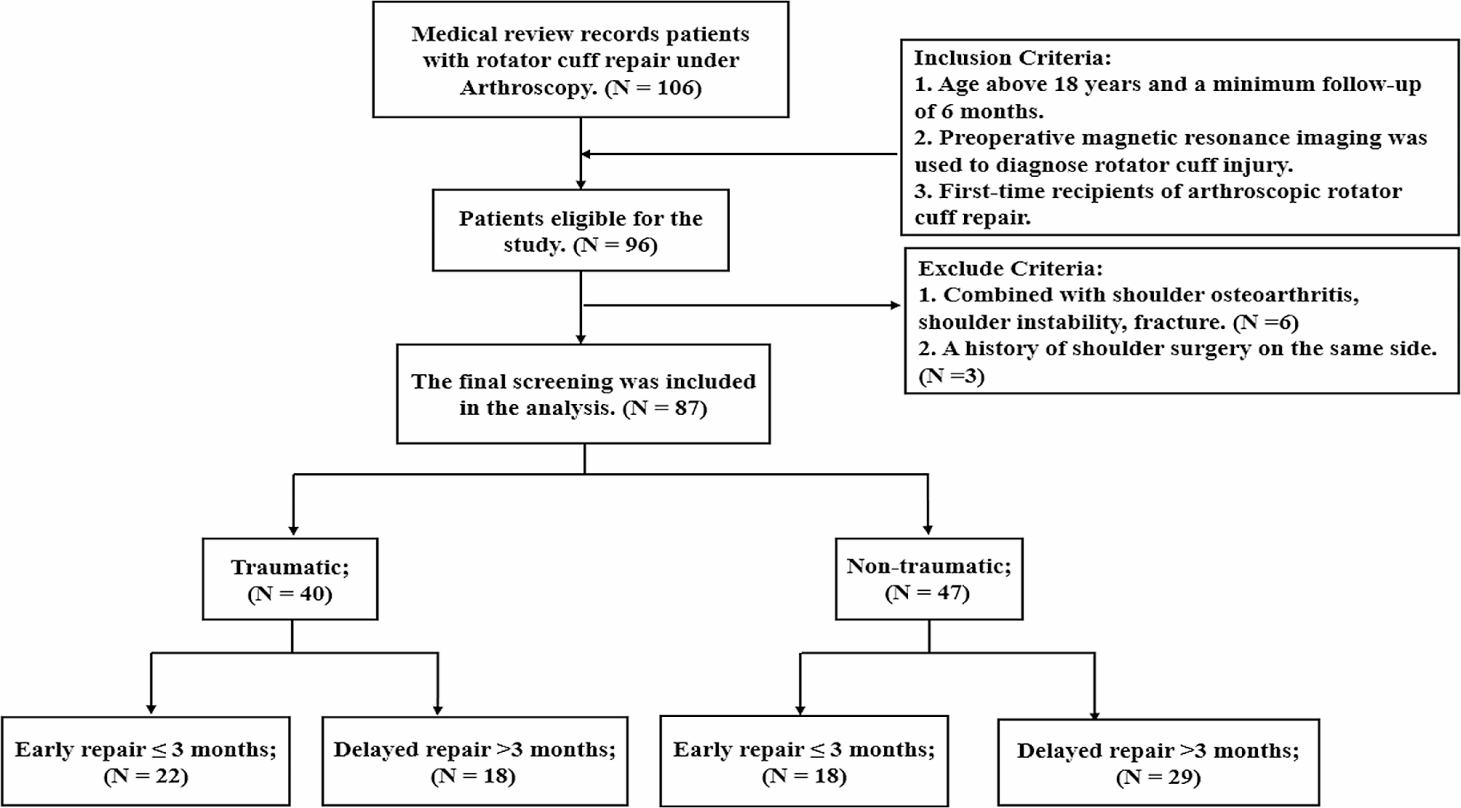
Flow diagram depicting the enrollment process for patients with rotator cuff injury

Ultimately, among the 106 rotator cuff injury patients whose charts were reviewed, 87 met the inclusion criteria (Fig. 2). Among patients with traumatic RCI, 22 patients underwent early repair, and 18 patients underwent delayed repair. Amongst patients with non-traumatic RCI, 18 patients underwent early repair, and 29 patients underwent delayed repair. In the traumatic group, gender (*p* = 0.751), age (*p* = 0.854), side of the affected limb (*p* = 0.737), the proportion of smokers (*p* = 0.731), hypertensive patients (*p* = 0.300), and diabetes patients (*p* = 0.680), as well as tear area (*p* = 0.409), duration of surgery (*p* = 0.546), type of repair (*p* = 0.748), number of full-layer tears (*p* = 0.750), and proportion of patients undergoing preoperative physical therapy (*p* = 0.185) and acromioplasty (*p* = 0.339) were comparable between the early and delayed repair groups. Similarly, in the non-traumatic group, gender (*p* = 0.760), age (*p* = 0.889), side of the affected limb (*p* = 0.869), the proportion of smokers (*p* = 0.673), hypertensive patients (*p* = 0.901), and diabetes patients (*p* = 0.788), as well as tear area (*p* = 0.775), duration of surgery (*p* = 0.742), type of repair (*p* = 0.948), number of full-layer tears (*p* = 0.345), and proportion of patients who received preoperative physical therapy (*p* = 0.055) and acromioplasty (*p* = 0.493) were comparable between the early and delayed repair groups (Table 1).

**Table 1:** Baseline characteristics of patients undergoing early and delayed repair in the trauma and non-trauma groups

	Early repair	Delayed repair	*P* Value
Traumatic *n* = 40			
Gender			0.751
Male, n (%)	12(54.5)	8(44.4)	
Female, n (%)	10(45.5)	10(55.6)	
Age, years	59.64 ± 6.84	59.28 ± 4.99	0.854
Left shoulder, n (%)	8(36.4)	5(27.8)	0.737
Right shoulder, n (%)	14(63.7)	13(72.2)	
Tobacco use, n (%)	5(22.7)	5(27.8)	0.731
Diabetes, n (%)	3(13.6)	4(22.2)	0.680
Hypertension, n (%)	4(18.2)	6(33.3)	0.300
Tear area, cm²	4.14 ± 1.19	3.86 ± 0.81	0.409
Duration of surgery, minutes	102.86 ± 14.33	100.39 ± 10.56	0.546
Repair technique, n (%)			0.748
Single row	8(36.4)	8(44.4)	
Double row	14(63.6)	10(55.6)	
Full layer tear, n (%)	13(59.1)	9(50.0)	0.750
Number of patients preoperative physiotherapy, n(%)	5(22.7)	8(44.4)	0.185
Number of acromioplasty patients, n (%)	8(36.3)	10(55.6)	0.339
**Nontraumatic***n* = 47			
Gender			0.760
Male, n (%)	7(38.9)	10(34.5)	
Female, n (%)	11(61.1)	19(65.5)	
Age, years	60.33 ± 6.48	60.62 ± 6.98	0.889
Left shoulder, n (%)	6(33.3)	9(31.0)	0.869
Right shoulder, n (%)	12(66.7)	20(69.0)	
Tobacco use, n (%)	4(22.2)	5(17.2)	0.673
Diabetes, n (%)	3(17.6)	4(13.8)	0.788
Hypertension, n (%)	4(22.2)	6(20.7)	0.901
Tear area, cm²	3.08 ± 0.38	3.12 ± 0.36	0.775
Duration of surgery, minutes	90.50 ± 13.11	91.72 ± 11.81	0.742
Repair technique, n (%)			0.948
Single row	7(38.9)	11(37.9)	
Double row	11(61.1)	18(62.1)	
Full layer tear, n (%)	5(27.8)	12(41.4)	0.345
Number of patients preoperative physiotherapy, n(%)	6(33.3)	18(62.1)	0.055
Number of acromioplasty patients, n (%)	12(66.7)	22(75.9)	0.493

### Imaging evaluation

In terms of involved tendons, there were no significant differences between the early repair subgroup and the delayed repair subgroup in the trauma and non-trauma groups (Table 2).

**Table 2:** Torn tendons of patients undergoing early and delayed repair in the trauma and non-trauma groups

	Early repair	Delayed repair	*P* Value
**Traumatic n = 40**			
1 tendon	6(27.3)	7(38.9)	0.509
Supraspinatus	5(22.7)	7(38.9)	0.315
Subscapularis	1(4.5)	0(0)	>0.99
2 tendons	15(68.2)	9(50.0)	0.335
Supraspinatus and infraspinatus	10(45.5)	6(33.3)	0.526
Supraspinatus and subscapularis	5(22.7)	3(16.7)	0.709
3 tendons: supraspinatus, infraspinatusand subscapularis	1(4.5)	2(11.1)	0.579
**Nontraumatic n = 47**			
1 tendon	10(55.6)	13(44.8)	0.474
Supraspinatus	9(50.0)	12(41.4)	0.563
Subscapularis	1(5.6)	1(3.4)	> 0.99
2 tendons	7(38.9)	14(48.3)	0.529
Supraspinatus and infraspinatus	5(27.8)	12(41.4)	0.345
Supraspinatus and subscapularis	2(11.1)	2(6.9)	0.631
3 tendons: supraspinatus, infraspinatusand subscapularis	1(5.6)	2(6.9)	> 0.99

### Functionality rating

**Fig. 3:**
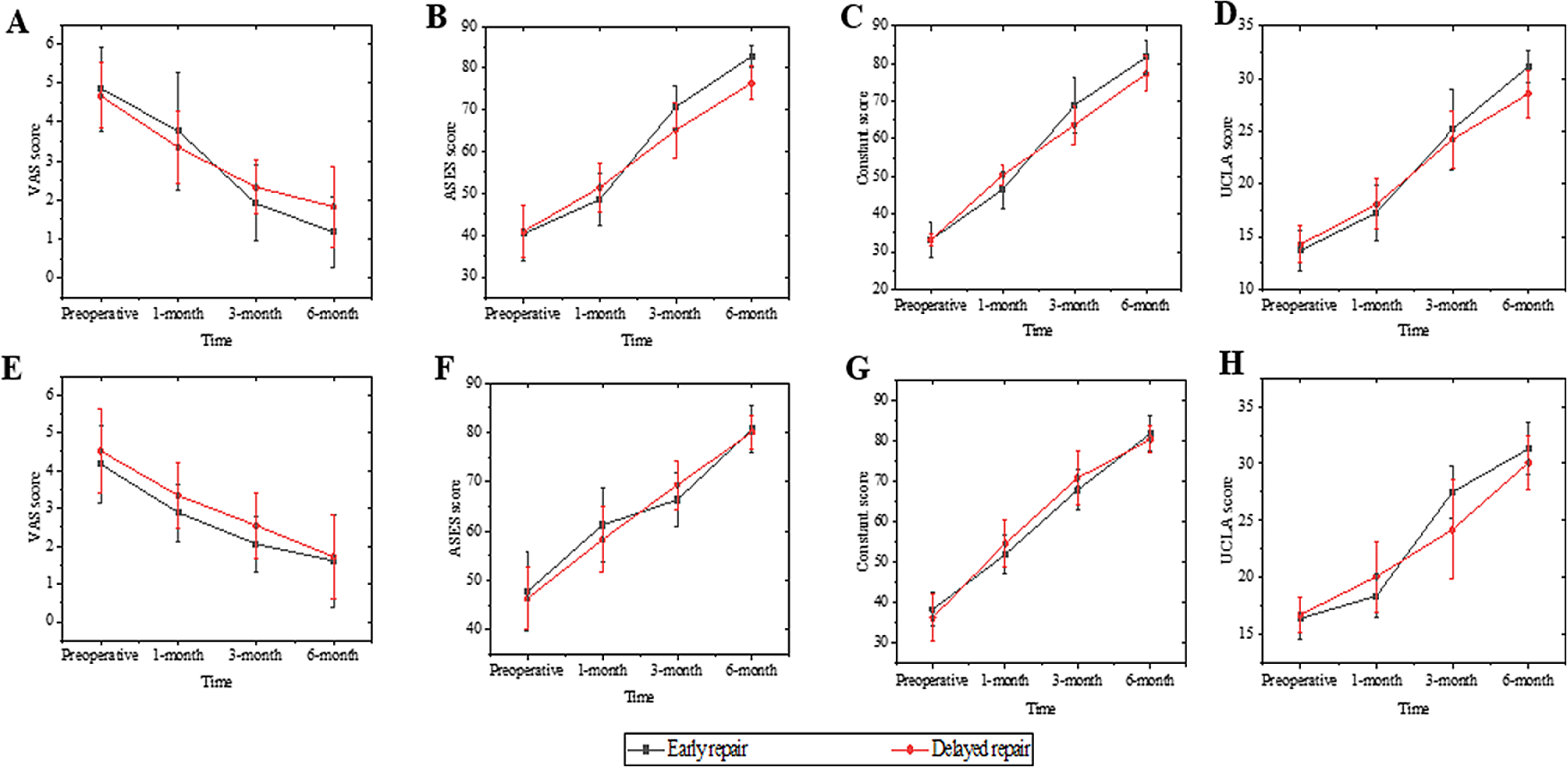
Comparison of visual analog scale (VAS) score, American Shoulder and Elbow Surgeons (ASES) score, Constant shoulder function score, and University of California Shoulder Score (UCLA) between patients undergoing early repair and delayed repair in the trauma and non-trauma groups. **A**-**D**, Comparison of functional scores between patients undergoing early and delayed repair in the trauma group. **E**-**H**, Comparison of functional scores between patients undergoing early and delayed repair in the non-trauma group. Early repair in the trauma group resulted in better functional outcomes at 6 months than delayed repair. In the non-trauma group, no significant difference between early and delayed repair 6 months postoperatively was observed

Preoperative VAS score (*p* = 0.531), ASES score (*p* = 0.787), Constant score (*p* = 0.948), and UCLA score (*p* = 0.316) were comparable between patients undergoing early and delayed repair in the trauma group. Likewise, there was no significant difference in preoperative VAS score (*p* = 0.291), ASES score (*p* = 0.504), Constant score (*p* = 0.205), or UCLA score (*p* = 0.528) between early and delayed repair patients in the non-trauma group. However, during postoperative follow-up, functional scores such as VAS scores (1.18 ± 0.91 vs. 1.83 ± 1.04, *p* = 0.041), ASES scores (82.89 ± 2.67 vs. 76.49 ± 4.00, *p* < 0.001), Constant scores (81.73 ± 4.62 vs. 77.28 ± 4.71, *p* = 0.005), and UCLA scores (31.14 ± 1.52 vs. 28.56 ± 2.25, *p* < 0.001) were higher in the trauma group of patients undergoing early repair than those undergoing delayed repair. On the other hand, during the follow-up of patients undergoing early and delayed repair in the non-trauma group, no statistically significant differences were observed in VAS scores (*p* = 0.746), ASES scores (*p* = 0.624), Constant scores (*p* = 0.259), and UCLA scores (*p* = 0.091) (Table 3; Fig. 3).

### Range of motion (ROM)

The range of motion of shoulder flexion (FF), external rotation (ER), and internal rotation (IR) were compared before and during follow-up across the 4 groups. The results revealed that the postoperative range of motion of patients undergoing early repair was superior to that of patients with delayed repair in the trauma group (FF, 149.55 ± 15.89 vs. 139.44 ± 12.10, *p* = 0.032; ER, 55.86 ± 5.93 vs. 49.44 ± 5.91, *p* = 0.002; IR, 41.36 ± 8.50 vs. 36.11 ± 6.98, *p* = 0.038). In comparison, early repair only outperformed delayed repair in terms of ER (55.56 ± 8.56 vs. 48.62 ± 14.26, *p* = 0.043) in the non-trauma group (Table 3).

### Postoperative complication

**Fig. 4:**
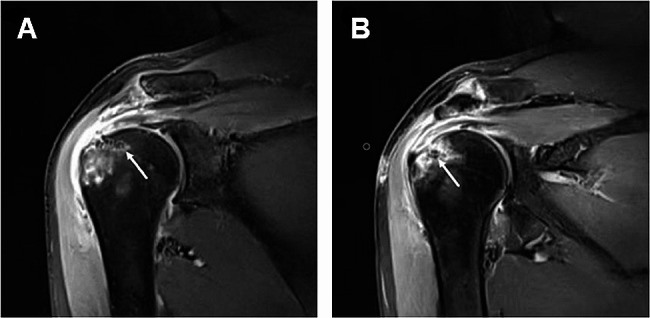
Postoperative follow-up of a 63-year-old patient with traumatic rotator cuff injury who underwent early repair, namely supraspinatus tendon repair and acromioplasty. **A** and **B** T2-weighted coronal magnetic resonance imaging illustrating the satisfactory positioning of the two anchors, partial healing of the supraspinatus tendon, and unresolved edema of the greater tuberosity of the humerus

During postoperative follow-up, 2 (9.1%) patients undergoing early repair and 3 (16.7%) patients undergoing delayed repair in the trauma group, as well as 2 (11.1%) patients undergoing early repair and 6 (20.7%) patients undergoing delayed repair in the non-trauma presented with joint stiffness; however, no statistically significant differences were noted between the two groups. Notably, these patients displayed improvements in joint stiffness during subsequent doctor-directed passive and active activities. At the same time, the incidence of rotator cuff re-tears was lower in patients undergoing early repair compared with those undergoing delayed repair in the trauma group (4.5% in 1 case vs. 33.3% in 6 cases, *p* = 0.033), whereas no statistical difference was noted in the non-trauma group (16.7% in 3 cases vs. 27.6% in 8 cases, *p* = 0.492) (Table 3; Fig. 4).

**Table 3:** Preoperative and postoperative results in patients undergoing early and delayed repair in the trauma and non-trauma groups

	Preoperative			Postoperative		
	Early repair	Delayed repair	*P* Value	Early repair	Delayed repair	*P* Value
**Traumatic n = 40**						
VAS score	4.86 ± 1.08	4.67 ± 0.84	0.531	1.18 ± 0.91	1.83 ± 1.04	0.041
ASES score	40.38 ± 6.56	40.93 ± 6.10	0.787	82.89 ± 2.67	76.49 ± 4.00	< 0.001
Constant score	33.18 ± 4.62	33.11 ± 1.75	0.948	81.73 ± 4.62	77.28 ± 4.71	0.005
UCLA score	13.68 ± 1.96	14.27 ± 1.74	0.316	31.14 ± 1.52	28.56 ± 2.25	< 0.001
ROM FF	87.41 ± 28.35	89.89 ± 24.91	0.773	149.55 ± 15.89	139.44 ± 12.10	0.032
ROM ER	44.77 ± 10.63	45.00 ± 8.23	0.941	55.86 ± 5.93	49.44 ± 5.91	0.002
ROM IR	24.55 ± 8.44	24.17 ± 8.27	0.887	41.36 ± 8.50	36.11 ± 6.98	0.038
Number of patients with joint stiffness, n (%)				2(9.1)	3(16.7)	0.642
Number of patients with rotator cuff retear, n (%)				1(4.5)	6(33.3)	0.033
**Nontraumatic n = 47**						
VAS score	4.17 ± 1.04	4.52 ± 1.12	0.291	1.61 ± 1.24	1.72 ± 1.10	0.746
ASES score	47.72 ± 7.96	46.31 ± 6.27	0.504	80.65 ± 4.79	80.06 ± 3.38	0.624
Constant score	38.33 ± 4.07	36.31 ± 5.84	0.205	81.61 ± 4.30	80.31 ± 3.44	0.259
UCLA score	16.44 ± 1.82	16.76 ± 1.53	0.528	31.28 ± 2.27	30.07 ± 2.37	0.091
ROM FF	102.78 ± 19.27	100.69 ± 20.73	0.732	145.83 ± 20.95	142.07 ± 24.29	0.590
ROM ER	48.61 ± 11.73	45.34 ± 13.88	0.411	55.56 ± 8.56	48.62 ± 14.26	0.043
ROM IR	31.94 ± 12.02	30.52 ± 6.99	0.651	42.22 ± 6.24	39.14 ± 8.46	0.188
Number of patients with joint stiffness, n (%)				2(11.1)	6(20.7)	0.692
Number of patients with rotator cuff retear, n (%)				3(16.7)	8(27.6)	0.492

VAS, Visual analogue scale; ASES, American shoulder and elbow surgeons; Constant, Constant-Murley; UCLA, The University of California, Los Angeles; ROM, range of motion; FF, forward flexion; ER, external rotation; IR, internal rotation

## Discussion

To the best of our knowledge, this is the first study to investigate differences in early and delayed repair among Chinese patients with both traumatic and non-traumatic RCI. Our findings exposed that surgical repair of traumatic RCI within 3 months post-injury yielded superior functional outcomes and patient satisfaction compared to repairs performed 3 months after injury. However, in the non-trauma group, early repair only demonstrated a significant improvement in external rotation of the shoulder compared to delayed repair, with no significant differences observed in other functional outcomes. The results of this study support our initial hypothesis that early repair of traumatic RCI results in better ROM and functional scores compared to delayed repair.

Arthroscopic rotator cuff repair has shown effectiveness in alleviating patient pain and improving function. A study examining [21] 188 arthroscopic repairs of isolated supraspinatus tendon tears reported that tendon healing alone did not fully account for subjective satisfaction among patients during follow-up beyond 1 year. This result may be ascribed to patients having high expectations regarding pain relief and functional improvement. Notably, in this study, patients in the trauma group who underwent early repair had superior outcomes, reporting higher subjective satisfaction scores on postoperative VAS, ASES, Constant, and UCLA scores compared to those undergoing delayed repair. Gutman [22] et al. examined 206 (150 males and 56 females) patients with traumatic rotator cuff tear repairs with a minimum of 24 months of postoperative follow-up. Interestingly, patients who underwent surgery within 4 months of injury achieved better functional recovery, consistent with the results of the present study. It is worthwhile emphasizing that Gutman et al. reported that patients who underwent repair within 2 to 4 months after injury were more likely to be young and had superior outcomes following tendon tear repair; this demographic difference may have influenced clinical outcomes post-surgery. Conversely, no statistical difference in age was observed between patients in the trauma and non-trauma groups who underwent early and delayed repair herein. Despite the diverse mechanisms underlying shoulder injuries, traumatic cases typically involve direct external forces acting on the shoulder joint, as evidenced by their greater tear size and pain scores [18]. Of note, a novel scoring system for assessing rotator cuff healing, pioneered by Kwon et al., implies that tear size is correlated with tendon involvement, with larger tear areas associated with higher postoperative re-tear rates [23]. Furthermore, a study undertaken by Lähteenmäki et al. suggested [24] that repairing a rotator cuff tear before it exceeds 2 cm² is associated with improved postoperative clinical outcomes, with a defined threshold of 2.5 cm². In the current study, patients in the trauma group undergoing early repair had tear areas of 4.14 ± 1.19 cm², while those undergoing delayed repair had tear areas of 3.86 ± 0.81 cm². Despite the higher tear size in patients undergoing early repair, their postoperative VAS, Constant, ASES, and UCLA scores were better than patients undergoing delayed repair. The improved postoperative functional scores may hold significant implications, considering the patients’ preoperative tear area and muscle degenerative changes.

Numerous studies have established [24, 25] the benefits of early surgical repair, particularly for traumatic tears, aiming to mitigate the detrimental effects of muscle atrophy and fat infiltration on the repair process. However, tailored treatments should be applied to each patient, taking into account their clinical conditions and comorbidities. While many physicians understand the difference between traumatic and non-traumatic causes of rotator cuff injuries, this disparity may not be well understood by novice rehabilitation therapists and patients. Consequently, it is crucial for healthcare professionals to identify the optimal timing for rotator cuff repair surgery. Following a traumatic event, some patients with traumatic injuries may postpone surgical intervention due to financial constraints or personal medical preferences, instead opting for conservative treatment. On the one hand, prolonged conservative management often results in unsatisfactory outcomes and delays the optimal window for repair. On the other hand, non-surgical protocols combined with appropriate physical therapy lead to better outcomes for patients with non-traumatic injuries [26, 27].

Studies focusing on the outcomes of non-operative treatment of non-traumatic rotator cuff injuries have reported conflicting results. Lambers Heerspink [28] et al. observed no difference in functional outcomes after 1 year of follow-up in a group of patients with non-traumatic rotator cuff injury who received either conservative treatment or surgical repair. In addition, Kuhn [7] et al. investigated 452 patients with nontraumatic rotator cuff injuries who were initially treated conservatively and showed satisfactory scoring results at weeks 6 and 12, yet a proportion (26%) of patients opted for surgery within 2 years of follow-up. Herein, patients in the non-trauma group typically experienced shoulder-associated symptoms for an average of 12.3 months before undergoing surgery, which was markedly longer than the trauma group. Most of these patients underwent an average of 3 months of conservative treatment before opting for surgery. Surprisingly, early surgical repair in the non-trauma group did not yield superior outcomes in terms of pain relief and functional improvement compared to delayed repair.

Tear size and chronicity, number of implicated tendons, level of fatty infiltration, and muscle atrophy have all been identified as significant indicators of functional outcome and repair integrity. Tear etiology (traumatic vs. atraumatic) may play a pivotal role in predicting functional outcomes. Moreover, patients with non-traumatic RCI are at a higher risk of developing muscle atrophy and fatty infiltration, which substantially influence post-repair functional outcomes. Earlier studies have uncovered that [25, 29] the average time from the onset of shoulder pain to surgical intervention in patients undergoing delayed repair in the non-trauma group was 13 months. However, muscular degradations span over the years. This prolonged timeframe is potentially a factor contributing to the lack of statistical differences in postoperative functional outcomes between patients undergoing early repair and delayed repair in the non-trauma group. An increasing number of experienced physicians recommend a trial of conservative treatment before considering surgical intervention for non-traumatic rotator cuff injuries. Additionally, they recommend surgical repair for cases not successfully managed by non-surgical approaches. Therefore, careful consideration of both non-operative and surgical treatment options is essential in the management of non-traumatic rotator cuff injuries.

Nevertheless, this study has several limitations that merit acknowledgment. To begin, this was a retrospective cohort study, and the findings were only able to establish correlation but not causation. Secondly, the mean age of traumatic injury patients included in this study was 59 years, and the possibility of asymptomatic degenerative tears being present before the trauma cannot be excluded, considering that the prevalence of asymptomatic tears is markedly higher in older adults than in younger adults [1, 5]. Thirdly, despite our rigorous screening of patients who experienced a traumatic event and our strict definition of “traumatic rotator cuff injury,” the subjective nature of trauma based on the mechanism of injury might have introduced potential bias. Fourthly, our study sample size was small, and the absence of preliminary power calculation; therefore, further studies with a large sample size are needed to confirm these results. Lastly, the follow-up period was relatively short, warranting further long-term assessments to evaluate the clinical outcomes of repair at various time points for both traumatic and non-traumatic rotator cuff injuries.

## Conclusions

In this study, early repair of traumatic rotator cuff injuries demonstrated superior outcomes in restoring range of motion, attenuating pain, and reducing the risk of postoperative re-tear compared to delayed repair. Therefore, early arthroscopic surgical repair remains the preferred approach for managing traumatic rotator cuff injuries. Conversely, for non-traumatic rotator cuff injuries, premature repair did not lead to improved clinical outcomes in postoperative joint function and complications. Consequently, early physiotherapy and non-surgical interventions are recommended as initial treatment approaches for patients with non-traumatic rotator cuff injuries. Taken together, these findings contribute to preoperative consultations regarding patient expectations and functional outcomes, considering tear etiology and timing of surgery, thereby enhancing our understanding of surgical outcomes.

## Data Availability

No datasets were generated or analysed during the current study.
